# Membrane fluidity properties of lipid-coated polylactic acid nanoparticles†

**DOI:** 10.1039/d3nr06464f

**Published:** 2024-04-01

**Authors:** Yuanqing Gu, Björn M. Reinhard

**Affiliations:** a Department of Chemistry and The Photonics Center, Boston University Boston MA 02215 USA yqgu@bu.edu; b Department of Chemistry and The Photonics Center, Boston University Boston MA 02215 USA bmr@bu.edu

## Abstract

Lipid coating is considered a versatile strategy to equip nanoparticles (NPs) with a biomimetic surface coating, but the membrane properties of these nanoassemblies remain in many cases insufficiently understood. In this work, we apply C-Laurdan generalized polarization (GP) measurements to probe the temperature-dependent polarity of hybrid membranes consisting of a lipid monolayer adsorbed onto a polylactic acid (PLA) polymer core as function of lipid composition and compare the behavior of the lipid coated NPs (LNPs) with that of liposomes assembled from identical lipid mixtures. The LNPs were generated by nanoprecipitation of the polymer in aqueous solutions containing two types of lipid mixtures: (i) cholesterol, dipalmitoylphosphatidylcholine (DPPC), and the ganglioside GM3, as well as (ii) dioleoylphosphatidylcholine (DOPC), DPPC and GM3. LNPs were found to exhibit more distinct and narrower phase transitions than corresponding liposomes and to retain detectable phase transitions even for cholesterol or DOPC concentrations that yielded no detectable transitions in liposomes. These findings together with higher GP values in the case of the LNPs for temperatures above the phase transition temperature indicate a stabilization of the membrane through the polymer core. LNP binding studies to GM3-recognizing cells indicate that differences in the membrane fluidity affect binding avidity in the investigated model system.

## Abstract 2

C-Laurdan generalized polarization (GP) measurements were applied to probe the temperature-dependent polarity of hybrid membranes consisting of a lipid monolayer adsorbed onto a polylactic acid (PLA) polymer core as function of lipid composition.

## Graphical abstract

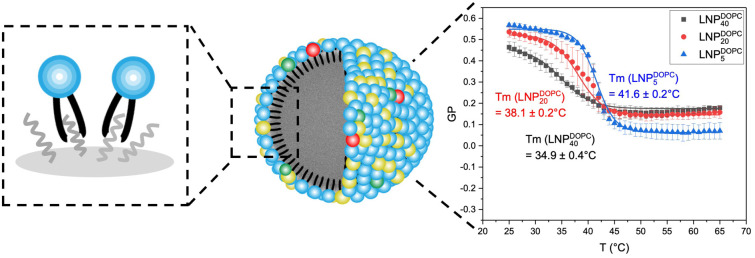

## Introduction

Lipid-coated nanoparticles (LNPs) contain a lipid membrane assembled around a central nanoparticle (NP) core. LNPs are versatile hybrid materials whose core can either be an inorganic “hard” NP, or a polymeric “soft” NP.^1–3^ LNPs with an inorganic core have found diverse applications, for instance as drug carriers (*e.g.* silica NP core^4–6^), in disease diagnosis and therapy (*e.g.* gold^7–9^ or iron oxide NP core^10–12^), or as biosensors (*e.g.* silver NPs^13^). Lipid-coated gold NPs have also been used as mimics to study the lipid-mediated cellular interactions of enveloped viruses^14^ and as nanoreactors^15^ that localize photoreactive species in the lipid coating around the core where they experience strong electrical (E-) field enhancements. Of particular interest for biomedical applications are, however, LNPs with a biodegradable polymeric core.^16–19^ The core can be loaded with pharmaceutical compounds or contrast agents, and the LNPs can serve as effective delivery agents or imaging probes. LNPs mimic enveloped viruses,^14^ and since their surface properties derive from the lipid coating, they can have fortuitous stealth properties. Most phospholipids, for instance, are zwitterionic which facilitates the assembly of LNPs with low affinity for non-specific biomolecule adhesion.^20,21^ Furthermore, bio-active lipids can also be utilized to provide LNPs with specific targeting functionalities. Ganglioside GM3 functionalized LNPs (GM3-LNPs), for instance, were shown to facilitate the selective targeting of CD169-myeloid cells and were developed into nanocarriers for antiretrovirals (ARVs) that target viral niches in macrophages and dendritic cells.^22,23^

Lipid-coated polymeric nanoparticles derive important surface properties from the lipid membrane, but the physicochemical properties of this membrane still pose important questions and may also depend on details of the fabrication. Two main strategies are applied to generate LNPs with a polymer core: the single step and the two-step assembly.^18,19^ In the two-step strategy, polymeric nanoparticles are generated first and then combined with pre-formed liposomes to form LNPs in which the polymeric core is encapsulated by a lipid bilayer membrane through direct hydration, sonication, or extrusion.^17–19,24,25^ In the single-step strategy LNPs are formed by nanoprecipitation of hydrophobic polymers in a lipid-containing aqueous solution or by emulsification–solvent–evaporation (ESE) in the presence of lipids as surfactants.^17–19,26^ Both single step strategies have in common that lipids form around the nascent core due to hydrophobic interactions between the hydrocarbon chains of the lipids and the polymer. Consequently, the lipid membrane formed under these conditions is expected to contain a monolayer of lipids that point with their hydrophobic tails towards the polymer core and with their hydrophilic head towards the aqueous phase.^7,8,13^ This membrane is best described as a hybrid membrane, and its structure and key properties, such as lipid packing and membrane fluidity require further investigation. Characterizing key properties of the hybrid membrane of LNPs is the focus of this study.

In the case of liposomes recent studies have shown that membrane fluidity is a factor that affects binding to^27^ and uptake^28^ by target cells. The potential effect of lipid order and membrane fluidity of the hybrid membrane on cell binding and uptake of LNPs is less clear. However, especially for membrane-embedded ligands that are weak monovalent binders, as in the case of GM3, and thus require multivalent interactions to increase the avidity,^23,29,30^ it stands to reason that the physical properties of the membrane affect the binding properties, for instance, through changes in the lateral mobility of the gangliosides. Structure and fluidity of membranes are frequently studied with polarity sensitive fluorescent probes, such as Laurdan or the structurally closely related but photophysically more stable C-Laurdan^31,32^ integrated into the membrane of interest. C-Laurdan is an amphiphilic molecule whose fluorescent emission spectrum is sensitive to the lipid packing in a membrane. The dye monitors changes in the polarity in the lipid membrane at the depth of the glycerol backbone due to changes in the packing of the lipids that modulate the water content in the membrane.^33,34^ The C-Laurdan emission peaks at 440 nm when the membrane is in a gel-phase with a relatively low concentration of water penetrating the membrane, and it shifts to 490 nm when the membrane is in a disordered fluid phase with a higher water content.^31,32,35^ Consequently, the generalized polarization (GP) defined by the following equation provides a measure for membrane polarity that correlates with membrane order and fluidity:1
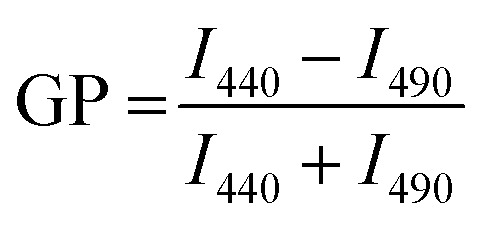


GP values, which are independent of the local probe concentration,^36^ range from −1 to 1, where high GP values indicate low polarity (*i.e.* high level of membrane order) and low GP values indicate high polarity (*i.e.* low level of membrane order). GP measurements have emerged as an effective experimental strategy to characterize membrane phase transitions in cellular membranes,^37^ liposomes^33,38,39^ and other hybrid vesicles.^40^ For liposomes GP values measured with the related Laurdan dye were found to distinguish between distinct phase states. Gel-phase, fluid-phase, and intermediate phase were assigned GP value ranges of GP > 0.55 (gel phase), GP < −0.05 (fluid phase), and −0.05 < GP < 0.55 (intermediate liquid-ordered and -disordered phases).^36^

**Fig. 1:**
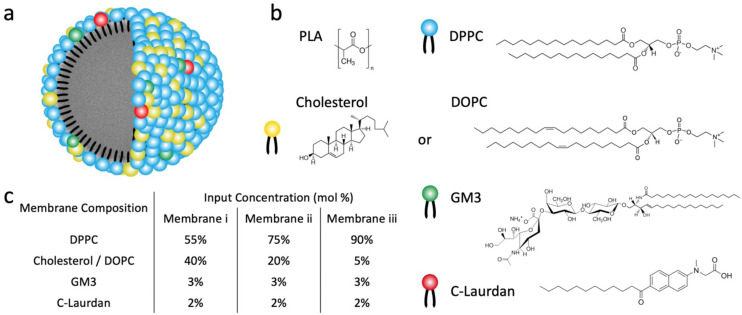
Structure and composition of GM3-LNPs and liposomes. (a) Schematic drawing of the GM3-LNP structure. (b) Chemical structure of the polymer core (PLA NP) and of membrane components (DPPC, Cholesterol/DOPC, GM3, C-Laurdan). (c) The input mol% of different lipid components for three different (i–iii) membranes of type **M1** (DPPC, cholesterol, GM3, C-Laurdan) and type **M2** (DPPC, DOPC, GM3, C-Laurdan) investigated in this work.

In this work, we apply C-Laurdan GP measurements to probe the polarity of hybrid membranes of different compositions self-assembled around a polylactic acid (PLA) polymer core. The hybrid membranes were assembled from mixtures of **M1**: saturated dipalmitoylphosphatidylcholine (DPPC), cholesterol, and small amounts of GM3, or **M2**: unsaturated dioleoylphosphatidylcholine (DOPC), DPPC, and small amounts of GM3 (Fig. 1a and b). These lipid compositions were chosen based on prior work with conventional liposomes^38,40–42^ and are anticipated to generate hybrid membranes with different levels of lipid packing and fluidity. Small amounts of GM3 were included in the lipid mix to characterize GM3-mediated binding to CD169-expressing cells as function of GP. In all experiments, liposomes of identical composition and similar size as the LNPs were included as benchmarks to facilitate a comparison of the LNP hybrid membrane properties with those of a conventional lipid bilayer. We found that LNPs have more distinct and narrower phase transitions than corresponding liposomes. Intriguingly, LNPs were found to exhibit detectable phase transitions even for cholesterol or DOPC concentrations that yielded no detectable transitions in liposomes. Furthermore, the GP values in the high temperature limit of all investigated GM3-LNPs were higher than those of liposomes with the same composition. Together these observations evidence that the presence of the polymer core decreases the polarity in the lipid layer surrounding the NPs when compared to conventional liposomes, indicating a higher packing density of hydrophobic chains, potentially induced by the interdigitation of polymer chain fragments and lipid tails in the hybrid membrane.

## Results and discussion

### Fabrication and characterization of GM3-LNPs and corresponding liposomes

**Fig. 2:**
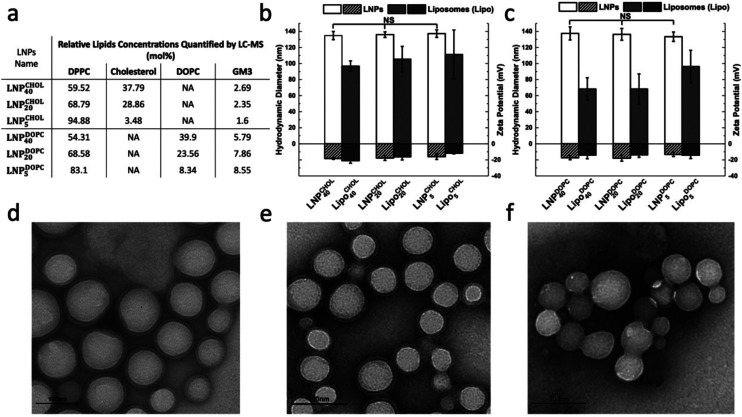
Characterization of GM3-LNPs and liposomes. (a) Relative lipids concentrations in GM3-LNPs quantified by LC-MS. (b and c) Hydrodynamic diameter and zeta potential of LNP^CHOL^_*x*_ and corresponding Liposome^CHOL^_*x*_ (Lipo^CHOL^_*x*_) (b), and of LNP^DOPC^_*x*_ and corresponding Liposome^DOPC^_*x*_ (Lipo^DOPC^_*x*_) (c). Error bars represent standard deviation. (d–f) TEM images of LNP^CHOL^_40_ (d), LNP^DOPC^_40_ (e), and PLA NPs (without lipid membrane) (f). Samples were treated with sodium phosphotungstate (1% w/v in water).

All LNPs used in this work were generated through dropwise addition of an organic solution of polylactic acid (PLA) (poly(d,l-lactide)), Resomer R207S, into an aqueous phase containing both lipids and C-Laurdan under stirring, followed by subsequent sonication. Liposomes were fabricated following established procedures^23^ through sonication of a re-hydrated lipid film that also contained C-Laurdan and subsequent extrusion. LNPs and liposomes generated with lipid mix M1 are in the following referred to as LNP^CHOL^_*x*_ and Liposome^CHOL^_*x*_ respectively, in which *x* stands for the input mol% of cholesterol in the lipid mix **M1**. Similarly, LNPs and liposomes generated with lipid mix **M2** are referred to as LNP^DOPC^_*x*_ and Liposome^DOPC^_*x*_, in which *x* means the input mol% of DOPC in the lipid mix M2. In all preparations the input concentration of GM3 was maintained at 3 mol%, and C-Laurdan was kept at 2 mol%. The input concentrations of cholesterol or DOPC were 5, 20, 40 mol%, respectively, and the rest of the lipid mix was made up by DPPC (90, 75, 55 mol%) (Fig. 1c). A total of six kinds of LNPs, namely LNP^CHOL^_5_, LNP^CHOL^_20_, LNP^CHOL^_40_, LNP^DOPC^_5_, LNP^DOPC^_20_, LNP^DOPC^_40_, and corresponding six kinds of liposomes, namely Liposome^CHOL^_5_, Liposome^CHOL^_20_, Liposome^CHOL^_40_, Liposome^DOPC^_5_, Liposome^DOPC^_20_, Liposome^DOPC^_40_ were investigated in this work. Given the complexity of the assembly process, it is unclear how the input concentration relates to the actual lipid ratios in the assembled membranes. Therefore, LC-MS was applied to measure the relative lipid concentrations in the assembled liposomes and LNPs (Fig. 2a). Overall, the measured lipid concentrations followed the input concentrations, but the measured ratios showed some deviations from the input ratios. Notably, the difference in cholesterol concentration between LNP^CHOL^_40_ and LNP^CHOL^_20_ was smaller than expected based on the input concentrations. This discrepancy was independently verified by a cholesterol fluorescence quantification assay (Fig. S1†). Furthermore, the GM3 concentrations in LNP^DOPC^_*x*_ were systematically higher than those in LNP^CHOL^_*x*_. Despite these deviations, the LC-MS data confirms the successful assembly of LNPs with systematic differences in cholesterol and DOPC content.

The size and *ζ*-potentials for LNP^CHOL^_*x*_ and Liposome^CHOL^_*x*_ are summarized in Fig. 2b, the corresponding data for LNP^DOPC^_*x*_ and Liposome^DOPC^_*x*_ are presented in Fig. 2c. For both LNP^CHOL^_*x*_ and LNP^DOPC^_*x*_, the average hydrodynamic diameters show no significant dependence on the lipid composition. The average hydrodynamic diameters are 136 ± 1 nm for LNP^CHOL^_*x*_ and 136 ± 2 nm for LNP^DOPC^_*x*_, with an average polydispersity index < 0.28. While LNP^CHOL^_*x*_ and LNP^DOPC^_*x*_ have essentially identically hydrodynamic diameters, the liposomes are smaller with average hydrodynamic diameters between 97 ± 7 and 111 ± 30 nm for Liposome^CHOL^_*x*_ and between 68 ± 14 and 96 ± 20 nm for Liposome^DOPC^_*x*_ with an average polydispersity index < 0.28. Due to its sialic acid GM3 carries a negative charge, and the fabricated LNPs and liposomes are negative with *ζ*-potentials in the range between −21 ± 3 mV to −12 ± 1 mV. Transmission electron microscopy (TEM) images of LNP^CHOL^_40_, LNP^DOPC^_40_ and PLA NPs (without lipid membrane) treated with sodium phosphotungstate Na_3_P(W_3_O_10_)_4_ are shown in Fig. 2d–f. To further verify the successful assembly of a lipid membrane around the polymer NP core, we colocalized LNPs and fluorescently labelled lipid membrane with an average colocalization percentage of 73% (Fig. S2†), which represents a conservative estimate of the fraction of lipid-coated NPs due to the rapid bleaching of the fluorescence. Consistent with the lipid-wrapped polymer NP design, the DSC thermograms (Fig. S3†) of LNP^CHOL^_20_ show two endothermic peaks, which correspond to the glass transition temperature of the polymer core and the phase transition temperature of the lipid membrane respectively.

### C-Laurdan GP measurement of LNP hybrid membranes and liposomes

**Fig. 3:**
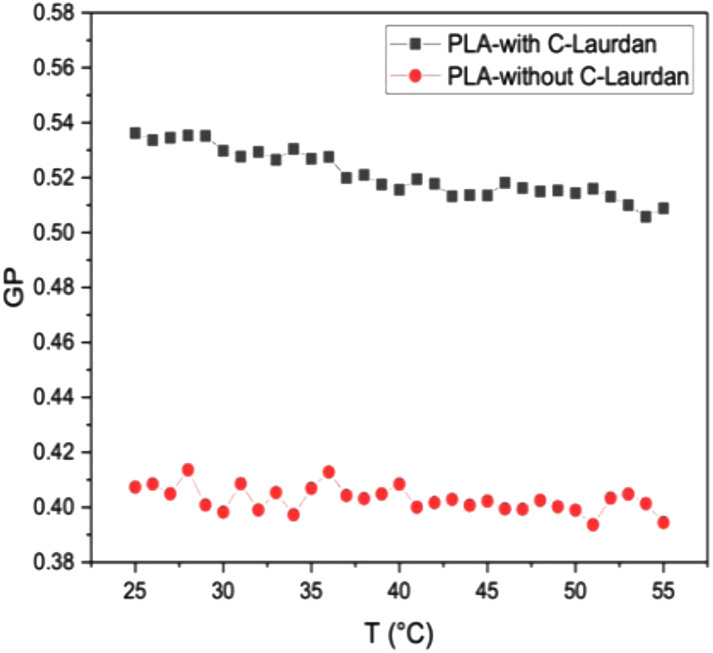
C-Laurdan GP measurements of PLA NPs (without lipid membrane). C-Laurdan GP(*T*) plots for PLA NPs without C-Laurdan (red circles), and PLA NPs with C-Laurdan (black squares).

In a first experiment we established baseline GP values for PLA NP controls generated by nanoprecipitation in a C-Laurdan containing solution in the absence of lipids. Fig. 3 contains the GP values of PLA NPs (no C-Laurdan) and PLA NPs with C-Laurdan in the temperature range between 25 °C to 55 °C. The GP values of C-Laurdan in PLA NPs are only slightly higher than for PLA NP background. Consistent with previous reports,^43,44^ the fluorescence signal of C-Laurdan in PLA NPs shows only a weak temperature dependence. In fact, the GP(*T*) curves for PLA NPs with C-Laurdan have nearly the same slope as the PLA controls without C-Laurdan and show a negligible decrease (ΔGP < 0.04) when compared with LNPs (Fig. S4†).

**Fig. 4:**
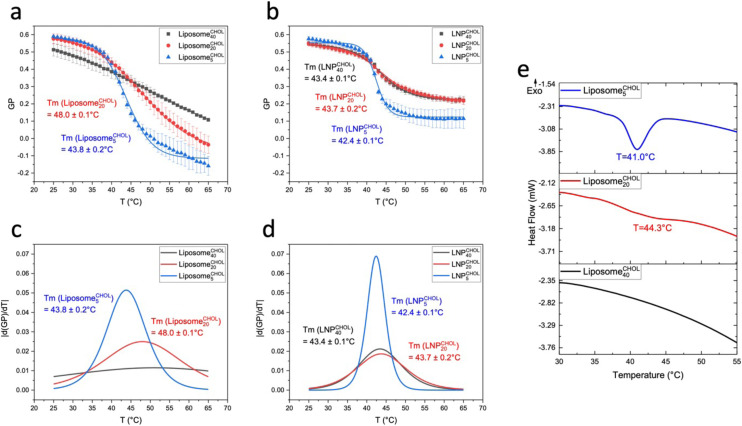
C-Laurdan GP(*T*) and DSC measurements of Liposome^CHOL^_*x*_ and LNP^CHOL^_*x*_. (a) GP(*T*) plots for Liposome^CHOL^_*x*_ (*n* = 3). (b) GP(*T*) plots for LNP^CHOL^_*x*_ (*n* = 4). Error bars in (a and b) represent standard deviation. (c) |d(GP)/d*T*| *vs. T* plots for Liposome^CHOL^_*x*_. (d) |d(GP)/d*T*| *vs. T* plots for LNP^CHOL^_*x*_. (e) DSC thermograms of Liposome^CHOL^_*x*_. *T* gives the phase transition temperature of the respective liposomes.

Next, we investigated the GP(*T*) relationships for LNP^CHOL^_*x*_ and Liposome^CHOL^_*x*_. Fig. 4a contains GP(*T*) plots for Liposome^CHOL^_5_, Liposome^CHOL^_20_, Liposome^CHOL^_40_ over the temperatures range between 25 °C to 65 °C. Fig. 4b shows equivalent data for LNP^CHOL^_*x*_ whose lipid coating was assembled from the same lipid mixture as liposomes. For both liposomes and LNPs the GP(*T*) curves show a much stronger decrease as function of temperature for all three investigated (hybrid) membrane compositions than observed for the PLA core. The decreasing sigmoidal GP(*T*) curves that are obvious for several experimental conditions indicate discrete phase transitions in the lipid membranes of liposomes and LNPs. All data were fitted to a Boltzmann sigmoidal^45^ (solid lines in Fig. 4a and b):2
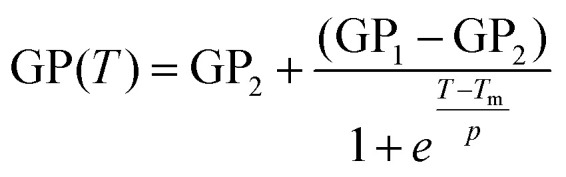


*T*_m_ represents an apparent thermal transition temperature of the membrane and corresponds to the temperature at which the GP value has dropped to 50% of its initial value.^37^ GP_1_ and GP_2_ are the low and high temperature asymptotic values of the GP(*T*) fit, and *p* accounts for the slope of the curve, which determines the width of the transition and is a measure of the cooperativity during the membrane phase transition. The absolute value of the first derivative of the decreasing sigmoidal function (|d(GP)/d*T*|) is a bell-shaped curve which allows for an easy determination of the onset (*T*_on_) and offset (*T*_off_) temperatures (Fig. 4c).^40^*T*_m_, *T*_on_, *T*_off_, Δ*T* between *T*_on_ and *T*_off_, GP values at 25 °C and 65 °C, ΔGP between 25 °C and 65 °C, as well as *p* values for Liposome^CHOL^_*x*_ and LNP^CHOL^_*x*_ are summarized in Table 1.

**Table 1:** The apparent thermal transition temperature (*T*_m_), GP values at 25 °C and 65 °C, ΔGP between 25 °C and 65 °C, *p* values, onset (*T*_on_) and offset (*T*_off_) temperatures of the apparent phase transition, Δ*T* between *T*_on_ and *T*_off_ for Liposome^CHOL^_*x*_ and LNP^CHOL^_*x*_

	*T*_m_ ( °C)	GP (25 °C)	GP (65 °C)	ΔGP(GP_*T*=25 °C_–GP_*T*=65 °C_)	*p*	*T*_on_ ( °C)	*T*_off_ ( °C)	Δ*T* (*T*_off_-*T*_on_) (°C)
Liposome^CHOL^_40_	NA	0.51 ± 0.04	0.11 ± 0.01	0.40 ± 0.05	17.59 ± 1.62	<25.0	>65.0	>40.0
Liposome^CHOL^_20_	48.0 ± 0.1	0.57 ± 0.03	-0.04 ± 0.05	0.61 ± 0.08	6.76 ± 0.12	27.6	>65.0	>38.0
Liposome^CHOL^_5_	43.8 ± 0.2	0.59 ± 0.01	-0.16 ± 0.05	0.75 ± 0.06	3.40 ± 0.11	31.0	57.0	26.0
LNP^CHOL^_40_	43.4 ± 0.1	0.55 ± 0.01	0.22 ± 0.02	0.33 ± 0.03	3.73 ± 0.17	29.8	57.5	27.7
LNP^CHOL^_20_	43.7 ± 0.2	0.55 ± 0.01	0.22 ± 0.02	0.33 ± 0.03	4.26 ± 0.19	29.1	59.3	30.2
LNP^CHOL^_5_	42.4 ± 0.1	0.57 ± 0.01	0.11 ± 0.06	0.46 ± 0.07	1.55 ± 0.12	35.9	49.0	13.1

The Liposome^CHOL^_5_ GP(*T*) curves indicates a distinct phase transition from gel (GP > 0.55) to liquid-crystalline phase (GP < −0.05) (Fig. 4a). For Liposome^CHOL^_20_ this transition is substantially broadened, and for Liposome^CHOL^_40_ the GP(*T*) has no longer a detectable sigmoidal character. The phase transition has been “washed out” and is no longer detectable. The changes in the GP(*T*) curves observed with GM3-containing liposomes for increasing cholesterol concentrations is consistent with differential scanning calorimeter (DSC) measurements performed with the same liposomes (Fig. 4e) as well as with previous Laurdan studies of the DPPC/cholesterol system and has been rationalized by the formation of a liquid-ordered phase throughout the investigated temperature range in the presence of medium to high concentrations of cholesterol.^38,41,46,47^ The observations that in the low temperature limit the GP values for Liposome^CHOL^_40_ lie lower than those of Liposome^CHOL^_5_, Liposome^CHOL^_20_, and that this order reverses at high temperatures reflects the well-known temperature dependence of the cholesterol effect on membrane fluidity, which means cholesterol decreases fluidity at high temperatures and increases fluidity at low temperatures.^48,49^

Unlike in the case of liposomes, the GP(*T*) curves for LNPs indicate discernible phase transitions for all investigated conditions (Fig. 4b). Even at high input concentrations of cholesterol, LNP^CHOL^_40_ still show a decreasing sigmoidal GP(*T*) behaviour, albeit with a smaller total change in GP. For all cholesterol concentrations, the fits yield *p* values whose absolute values are smaller than those of liposomes with identical composition, which is consistent with narrower transitions (*i.e.* a small temperature differences (Δ*T*) between *T*_on_ and *T*_off_) (Fig. 4c, d and Table 1) due to a higher degree of cooperativity. The recorded GP values of LNP^CHOL^_5_ indicate transitions from a gel phase (GP = 0.57 ± 0.01) to liquid-disordered intermediate phase (GP = 0.11 ± 0.06). For LNP^CHOL^_20_ and LNP^CHOL^_40_, GP values of 0.22 ± 0.02 are measured in the high temperature limit, suggesting a state between liquid-ordered and liquid-disordered phase. The measured phase transition temperatures *T*_m_ for LNPs with different cholesterol content are very similar with a maximum difference of 1.3 °C. For Liposome^CHOL^_*x*_ an increase in *T*_m_ of 4.2 °C is recorded between Liposome^CHOL^_5_ and Liposome^CHOL^_20_, indicating a stronger cholesterol dependence of *T*_m_ in liposomes than in LNPs.

**Fig. 5:**
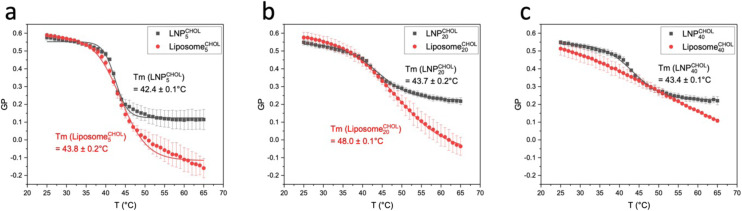
Comparison of GP(*T*) curves of LNP^CHOL^_*x*_ and Liposome^CHOL^_*x*_ with the same cholesterol content. GP(*T*) curves of LNP^CHOL^_*x*_ and Liposome^CHOL^_*x*_ with *x* = 5 (a), *x* = 20 (b), and *x* = 40 (c). Error bars represent standard deviation.

**Fig. 6:**
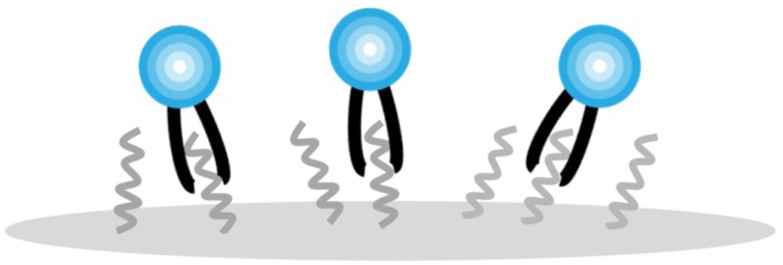
Schematic representation of the interdigitation between hydrophobic polymer chains and surrounding lipid tails.

Fig. 5a–c compares GP(*T*) curves of Liposome^CHOL^_*x*_ and LNP^CHOL^_*x*_ with identical membrane composition. In the low temperature limit, the GP values for liposomes and LNPs are nearly identical except Liposome^CHOL^_40_ and LNP^CHOL^_40_, for which the GP values of the LNP^CHOL^_40_ lie slightly higher than those of Liposome^CHOL^_40_. In the high temperature limit, the GP values of LNPs lie overall higher than those of liposomes. However, since the GP values of liposomes also show a more pronounced increase as function of increasing cholesterol input, the gap between the high temperature GP limits of liposomes and LNPs narrows with increasing cholesterol concentration in the high temperature limit. The increase in GP with increasing cholesterol content in liposomes for *T* > *T*_m_ indicates an increasing membrane order that can be understood in terms of the established cholesterol condensing effect that results in a closer packing of lipids.^48,49^ It is also interesting that the high temperature GP values for LNP^CHOL^_5_ are similar to those of Liposome^CHOL^_40_. This observation implies that the polymer core of the LNPs has a similar effect on the polarity of the lipid layer as cholesterol in liposomes in the high temperature range. We attribute this behaviour to an interdigitation of hydrophobic polymer chains into the surrounding lipid layer (Fig. 6). These additional polymer segments increase the density of hydrophobic components in the resulting hybrid membrane, and it is conceivable that hydrophobic interactions between polymer segments and lipid tails favour a parallel alignment that result in an increased order and tighter packing. A similar interpretation was previously presented for the rigidification of a lipid monolayer around cross-linked polysaccharide NPs functionalized with long chain (C16) fatty acids by Peyrot *et al.*^50^ Stabilizing hydrophobic interactions between lipid tails and surface polymer chains could also explain the weak cholesterol dependence of *T*_m_ observed for LNPs.

**Fig. 7:**
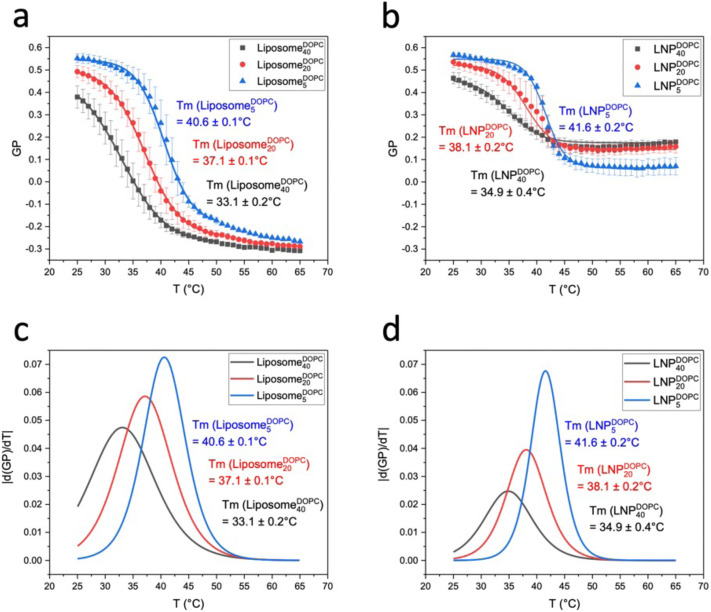
C-Laurdan GP(*T*) measurements of Liposome^DOPC^_*x*_ and LNP^DOPC^_*x*_. (a) C-Laurdan GP(*T*) plots for Liposome^DOPC^_*x*_ (*n* = 3). (b) C-Laurdan GP(*T*) plots for LNP^DOPC^_*x*_ (*n* = 4) Error bars in (a and b) represent standard deviation. (c) |d(GP)/d*T*| *vs. T* plots for Liposome^DOPC^_*x*_. (d) |d(GP)/d*T*| *vs. T* plots for LNP^DOPC^_*x*_.

After characterizing GP(*T*) curves for Liposome^CHOL^_*x*_ and LNP^CHOL^_*x*_, we will now turn our attention to GP(*T*) curves for liposomes and LNPs with DOPC input concentrations of 5, 20, and 40 mol% (Fig. 7a and b). Liposomes and LNPs exhibited decreasing sigmoidal GP(*T*) curves that were well described by eqn (2) for all DOPC concentrations. Fig. 7c and d contains plots of the first derivative of GP *versus* the temperature (|d(GP)/d*T*|). *T*_m_, *T*_on_, *T*_off_, Δ*T* between *T*_on_ and *T*_off_, GP values at 25 °C and 65 °C, ΔGP between 25 °C and 65 °C, as well as *p* values are summarized in Table 2. LNPs and liposomes with identical DOPC content show comparable transition temperatures, *T*_m_, which systematically decrease with increasing DOPC concentration due to increasing membrane disorder.

**Table 2:** The apparent thermal transition temperature (*T*_m_), GP values at 25 °C and 65 °C, ΔGP between 25 °C and 65 °C, *p* values, onset (*T*_on_) and offset (*T*_off_) temperatures of the apparent phase transition, Δ*T* between *T*_on_ and *T*_off_ for Liposome^DOPC^_*x*_ and LNP^DOPC^_*x*_

	*T*_m_ (°C)	GP (25 °C)	GP (65 °C)	ΔGP(GP_*T*=25 °C_–GP_*T*=65 °C_)	*p*	*T*_on_ (°C)	*T*_off_ (°C)	Δ*T*(*T*_off_-*T*_on_) ( °C)
Liposome^DOPC^_40_	33.1 ± 0.2	0.38 ± 0.05	−0.31 ± 0.00	0.69 ± 0.05	3.93 ± 0.09	<25.0	48.9	>23.9
Liposome^DOPC^_20_	37.1 ± 0.1	0.49 ± 0.03	−0.29 ± 0.00	0.78 ± 0.03	3.21 ± 0.07	<25.0	50.3	>25.3
Liposome^DOPC^_5_	40.6 ± 0.1	0.55 ± 0.02	−0.27 ± 0.00	0.82 ± 0.02	2.53 ± 0.09	30.6	51.4	20.8
LNP^DOPC^_40_	34.9 ± 0.4	0.46 ± 0.03	0.18 ± 0.00	0.28 ± 0.03	2.88 ± 0.32	<25.0	46.6	>21.6
LNP^DOPC^_20_	38.1 ± 0.2	0.54 ± 0.02	0.16 ± 0.03	0.38 ± 0.05	2.39 ± 0.13	29.0	47.9	18.9
LNP^DOPC^_5_	41.6 ± 0.2	0.57 ± 0.01	0.07 ± 0.04	0.50 ± 0.05	1.80 ± 0.12	34.1	49.3	15.2

The measured GP values of Liposome^DOPC^_5_ indicate a transition from gel phase (GP = 0.55 ± 0.02) to fluid phase (GP = −0.27). The GP values of liposomes in the low temperature limit decrease with increasing DOPC concentration. The GP (*T* = 25 °C) values for Liposome^DOPC^_20_ and Liposome^DOPC^_40_ are 0.49 ± 0.03 and 0.38 ± 0.05, respectively. These data evidence an increase in average membrane polarity in the low temperature limit due to a decrease of the average lipid packing density with increasing concentration of unsaturated lipid. Previous studies have shown that a binary DOPC/DPPC mix exhibits a gel – liquid crystalline coexistence region over a broad DOPC concentration range at 25 °C.^42^ The decrease in GP observed for increasing DOPC concentration is consistent with a gradual increase in the contribution from the liquid crystalline phase in the coexistence region. In the high temperature limit, the differences in the GP values for liposomes with different DOPC concentrations are less distinct and the GP(*T*) curves converge.

**Fig. 8:**
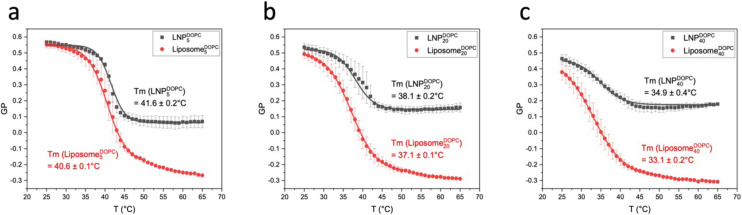
Comparison of GP(*T*) curves of LNP^DOPC^_*x*_ and Liposome^DOPC^_*x*_ with the same DOPC content. GP(*T*) curves of LNP^DOPC^_*x*_ and Liposome^DOPC^_*x*_ with *x* = 5 (a), *x* = 20 (b), and *x* = 40 (c). Error bars represent standard deviation.

LNP^DOPC^_*x*_ also show a decrease in GP values with increasing DOPC content in the low temperature limit, but the GP values for *T* = 25 °C do not drop below GP = 0.45 for LNP^DOPC^_40_. In the high temperature limit the GP(*T*) curves for LNPs lie systematically higher than for liposomes (Fig. 8). The GP (*T* = 65 °C) values are negative for liposomes but positive for LNPs. There are also smaller differences between LNPs with different DOPC concentrations in the high temperature limit; the GP values for LNP^DOPC^_5_ lie systematically lower than those of LNP^DOPC^_20_ or LNP^DOPC^_40_, but the difference is small (∼0.1 unit). Overall, the lower polarity of the hybrid membranes in the high temperature limit as indicated by higher GP values for LNPs suggests that the phase transition in the hybrid membrane of DOPC-containing LNPs is associated with a less distinct structural change than in DOPC liposomes. We again attribute this difference to an interdigitation of polymer chain fragments and self-assembled lipid monolayer in the case of the LNPs. Lateral hydrophobic interactions between polymer chain fragments and both saturated and unsaturated lipids can stabilize the hybrid membrane and decrease the change in membrane polarity associated with the phase transition. The polymer chain fragments represent a framework that impedes lateral lipid motion, thus limiting the fluidity of the hybrid membrane at elevated temperatures.

### GM3-CD169-mediated binding of GM3-LNPs to Raji B CD169^+^ cells

**Fig. 9:**
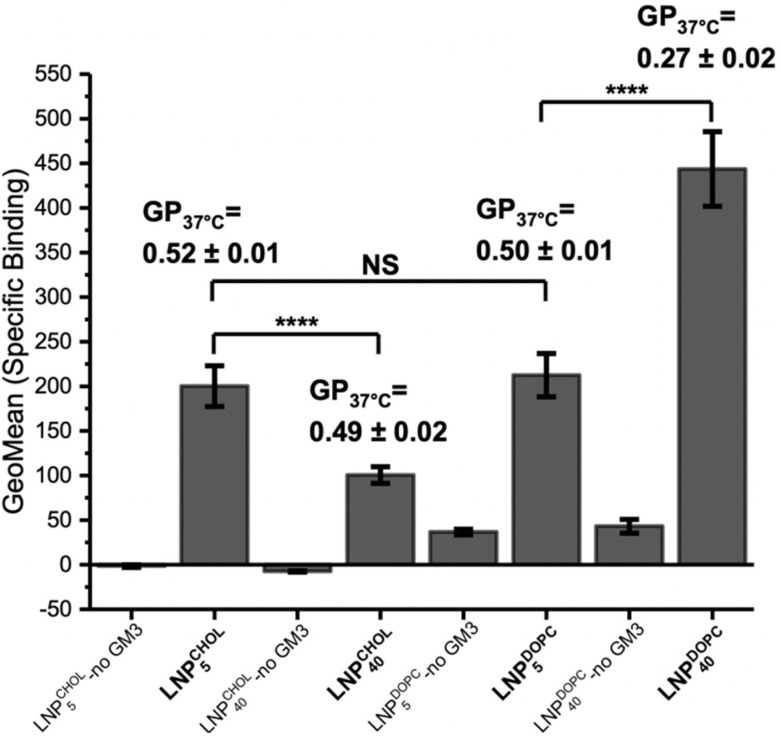
Binding of different GM3-LNPs to CD169^+^ Raji B cells. From left to right: LNP^CHOL^_5_ without GM3, LNP^CHOL^_5_, LNP^CHOL^_40_ without GM3, LNP^CHOL^_40_, LNP^DOPC^_5_ without GM3, LNP^DOPC^_5_, LNP^DOPC^_40_ without GM3, LNP^DOPC^_40_. The GP (*T* = 37 °C) are included for LNP^CHOL^_5_, LNP^CHOL^_40_, LNP^DOPC^_5_, and LNP^DOPC^_40_ (*n* = 10).

GM3-LNPs are interesting drug delivery platforms as they combine GM3-mediated targeting of CD169-expressing cells with the programmable release properties of the PLA core.^22^ Individual GM3-CD169 contacts are relatively weak with a dissociation constant *K*_D_ in the mM range, but multivalent presentation can result in an increase in avidity.^51,52^ Differences in the mobility of GM3 in the membrane of liposomes or LNPs could affect collective binding interactions and thus avidity.^29^ In this context it is therefore relevant that the measured GP values for DOPC liposomes and LNPs indicate differences in lipid packing and fluidity at 37 °C. These differences may affect GM3-mediated binding to CD169-expressing cell surfaces. Fig. 9 compares the specific GM3 binding of LNP^CHOL^_5_ and LNP^CHOL^_40_, as well as LNP^DOPC^_5_ and LNP^DOPC^_40_ to CD169^+^ Raji B cells as determined by flow cytometry. In all experiments, the LNP concentration was monitored by UV-Vis measurements and kept constant, and LNPs without GM3 (the DPPC content was increased to account for the lack of GM3) were included as controls. All GM3-containing LNPs showed much more binding than the LNP controls, confirming that binding is dominated by GM3-CD169 interactions. Consistent with this interpretation, GM3-LNPs showed negligible binding to CD169^−^ Raji B cells (Raji B cells which did not express CD169). Intriguingly, LNP^DOPC^_40_ which have a lower GP and thus more fluid membrane than all other investigated GM3-LNPs, show significantly more binding to CD169^+^ Raji B cells than any other experimental condition. This is especially remarkable considering that – according to our LC-MS measurements in Fig. 2a – the GM3 content of LNP^DOPC^_40_ was lower than for LNP^DOPC^_5_ or LNP^DOPC^_20_. The observed increase in binding for GM3-LNPs with the lowest GP value is indicative of a gain in binding avidity and corroborates the hypothesis that increased membrane fluidity benefits GM3-CD169-mediated cell binding.

## Conclusions

This work has investigated the temperature-dependent polarity of hybrid membranes around a PLA core using the fluorescent C-Laurdan probe. In the low temperature limit (*i.e.* at room temperature) LNP^CHOL^_*x*_ and LNP^DOPC^_*x*_ were found to have GP values that are comparable to those of liposomes with identical membrane composition. However, consistently higher GP values for LNPs than for liposomes at elevated temperatures evidence that LNPs contain the C-Laurdan probe located in an environment that is less polar than in the corresponding liposomes. One model to account for this gain in polarity for the LNP hybrid membrane is a structure that contains an outer lipid layer at least partially interdigitating into an inner layer of polymer chain segments that define the surface of the PLA NP. The polymer segments of the PLA core structure the lipid layer through lateral hydrophobic interactions and reduce the fluidity of the lipid membrane. Due to this structure-stabilizing effect, which was observed for both saturated and unsaturated lipids, LNPs retain detectable phase transitions even for membrane compositions that lack detectable phase transitions in liposomes. The fluidity of the hybrid membrane of GM3-LNPs was found to increase GM3-CD169-mediated cell binding under otherwise identical conditions, underlining the potential for controlling lipid-mediated binding through rationally designed LNPs.

## Materials and methods

### Materials

All chemicals were used as received. 1,2-dipalmitoyl-*sn-glycero*-3-phosphocholine (DPPC), 1,2-dioleoyl-*sn-glycero*-3-phosphocholine (DOPC), cholesterol, GM3 ganglioside (Milk, Bovine-Ammonium Salt) and 1,2-dipalmitoyl-*sn-glycero*-3-phosphoethanolamine-*N*-(lissamine rhodamine B sulfonyl) (ammonium salt) (16 : 0 Liss Rhod PE) were purchased from Avanti Polar Lipids Inc. *N*-Methyl-*N*-[6-(1-oxododecyl)-2-naphthalenyl]glycine (C-Laurdan) was purchased from R&D Systems Inc. Chloroform, methanol, acetonitrile (all HPLC grade), sodium phosphotungstate hydrate, and ester terminated polylactic acid (PLA) (poly(d,l-lactide)), Resomer R207S,^53^ with a mass-average molecular weight (*M*_W_) of 262 000 g mol^−1^ and polydispersity of 1.6 were purchased from Sigma–Aldrich Inc.

### Methods

#### Preparation of lipid-wrapped polymeric nanoparticles

Lipid-coated polymeric nanoparticles (LNPs) were prepared through one-step nanoprecipitation synthesis, as described previously.^23,54^ A total lipid amount of 0.25 μmole,^54^ an aqueous/organic solution volume ratio of 10 : 1^55^ and a lipid/polymer weight ratio of 15%^26^ was chosen for the synthesis. A lipid mixture containing DPPC (25 mg mL^−1^ in chloroform), cholesterol (25 mg mL^−1^ in chloroform), GM3 (2 mM in methanol/chloroform (1 : 1)), and C-Laurdan (1 mM in chloroform) (M1) or DPPC (25 mg mL^−1^), DOPC (25 mg mL^−1^ in chloroform), GM3 (2 mM), and C-Laurdan (1 mM) (M2) with mol% as-specified in the text was added to around 4 mL of Milli-Q water. Next, around 0.4 mL of high molecular weight PLA solution in acetonitrile (2.5 mg mL^−1^) was pipetted dropwise to the above aqueous solution under stirring. The final solution was vortexed for 10 s and then sonicated in a bath sonicator (Branson Ultrasonics, No. 5510, Danbury, CT) for 6 min. Finally, NPs were washed 3 times (4500 g, 15 min) using 10 kDa Amicon Ultra-4 centrifugal filter (Millipore Sigma, Burlington, MA) to remove organic solvent and free lipid molecules. Polymer NPs without membrane (PLA NPs) were obtained following the same procedure in the absence of lipids but with C-Laurdan. DLS was applied to measure the diameter of the samples. 2 mol% of fluorescent lipid (16 : 0 Liss Rhod PE) instead of C-Laurdan was incorporated to all LNPs for labelling when LNPs were subjected to flow cytometry. The different LNP preparations used in the cell binding experiments had essentially identical diameters. Assuming identical dye concentrations in the lipid coating, UV-Vis absorbance measurements were used to ensure identical concentrations for the different LNP preparations in the binding studies.

#### Fabrication of liposomes

Liposomes were formulated according to the Bangham method^56^ with certain adjustments. A lipid mixture containing DPPC (10 mM), cholesterol (10 mM), GM3 (2 mM), C-Laurdan (1 mM) (M1) or DPPC (10 mM), DOPC (10 mM), GM3 (2 mM), C-Laurdan (1 mM) (M2) in chloroform with mol% as-specified in the text was added to a 25 mL round-bottom flask. The total lipid amount was maintained at 1 μmole. Then, the solvent was removed by rotary evaporation (34 °C, 10 min) to obtain a homogeneous and thin lipid film and the samples were dried overnight under vacuum. Multilamellar vesicles (MLVs) were obtained by adding 1 mL of Milli-Q water to this lipid dry film and then sonicating for 5 min using a probe sonicator (120 Sonic Dismembrator, Fisher Scientific, Waltham, MA). Finally, a dispersion of small unilamellar vesicles (SUVs) was obtained by extruding the obtained MLV dispersion 6 times through a calibrated polycarbonate membrane with a pore diameter of 100 nm using the Avanti® mini extruder (Avanti Polar Lipids Inc).

#### Dynamic light scattering (DLS) and zeta-potential measurements

Hydrodynamic size and zeta-potential of LNPs and liposomes were measured by Zetasizer Nano ZS90 (Malvern, Worcestershire, UK) at room temperature. For hydrodynamic size measurements, NPs were diluted with Milli-Q water. The zeta potential was determined in 10 mM NaCl solution.

#### Transmission electron microscopy (TEM) characterization

To prepare samples for inspection in TEM, lipid-coated polymeric nanoparticles (LNPs) and polymer NPs without membrane (PLA NPs) NPs were drop-cast onto carbon-coated TEM grids and incubated for 20 min before removing the excess solution by a clean filter paper. Next, samples were stained with 1% sodium phosphotungstate Na_3_P(W_3_O_10_)_4_ in water (w/v) for 10 s and excess stain solution was removed. Samples were dried and stored under vacuum before imaging using a Hitachi 7800 TEM (HT7800) with 100 kV acceleration voltage.

#### Quantification of relative lipids concentrations

Lipids were extracted from lipid-coated polymer nanoparticles (LNPs) through a mixture of chloroform–methanol (1 : 1, v/v), and a single-phase solution was obtained after stirring overnight. LC-MS analysis was performed by the Harvard Center for Mass Spectrometry. All samples were run on a Thermo Orbitrap QE+/Ultimate 3000 LC (ThermoFisher Qexactive Plus) with source HESI+, full MS 70k resolution, 3 × 10^6^ target AGC, mz 300–1500, max IT 100 ms. A Dikma biobond C4 (50 × 4.6 mm) column was used with column temperature at 45 °C, sample temperature at 4 °C, and injection volume of 5 μL.

#### C-Laurdan generalized polarization (GP) measurements

Steady-state fluorescence measurements with C-Laurdan were performed with a spectrofluorometer (Horiba, Piscataway, NJ) equipped with temperature control, using 1 cm path length quartz cuvettes. C-Laurdan emission spectra (420–510 nm) of LNPs and liposomes were collected from 25 °C to 65 °C at an interval of 1 °C with an equilibration time of 0.5 min at each temperature upon excitation at 405 nm. C-Laurdan GP values at different temperature were calculated using the difference in emission intensities at 440 and 490 nm according to eqn (1) and GP(*T*) plots were obtained. Note that background intensities of LNPs and liposomes without C-Laurdan were minimal and negligible (Fig. S5†), so only the fluorescence intensities of the samples containing C-Laurdan without background subtraction were recorded for GP measurements. The decreasing sigmoidal GP(*T*) curve was fitted to a Boltzmann sigmoidal function (eqn (2)) to derive the apparent thermal transition temperature (*T*_m_) of the membrane, low (GP_1_) and high (GP_2_) temperature asymptotic values, and *p* values which show the cooperativity during the membrane phase transition. To assess the onset (*T*_on_) and offset (*T*_off_) temperatures, the absolute value of the first derivative with respect to temperature of the decreasing GP(*T*) sigmoidal function was further evaluated, which showed a bell-shaped curve with baseline values corresponding to the small slopes of the GP(*T*) curves. The points at which the slope begins to deviate sharply from and returns to its baseline values (*i.e.* the start and the end of the peak) correspond to *T*_on_ and *T*_off_. To determine these points, tangent lines were constructed along the peaks and representative tangent constructions are shown in the Fig. S6.†*T*_on_ and *T*_off_ were taken to be the averages of the intersection of two left-side tangents and two right-side tangents with the baseline of each peak respectively, analogous to the procedure followed to find transition onset and completion temperatures using differential scanning calorimetry (DSC).^40^

#### Differential scanning calorimetry (DSC) measurements

To characterize thermal behaviour of LNPs and liposomes, 20 μL of concentrated solution was transferred into an aluminum pan (Thermal Support, Hayesville, NC) and sealed. Thermograms from 30 °C to 55 °C, at a heating rate of 5 °C min^−1^ were recorded by using a Mettler Toledo Polymer DSC R (Mettler-Toledo, Columbus, OH). The DSC software integrated into the instrument was used to determine the phase transition temperatures.

#### Determination of NP concentrations through UV-Vis

The absorption spectra of LNPs in Milli-Q water were acquired using a Spectronic 200 UV-Vis spectrometer (Fisher Scientific, Waltham, MA) and Milli-Q water was used for baseline correction. Beer's law was used to calculate the concentration of LNPs using the absorption value of fluorescently labelled lipid (16 : 0 Liss Rhod PE) at the wavelength of 570 nm (Lambda max), and the molar absorptivity of *ε* = 73 000 M^−1^ cm^−1^.^23^ The final volume of samples was adjusted to 100 μL with a typical concentration of 10^12^ NPs mL^−1^.

#### Cell culture

Raji B cells were cultured in complete RPMI-1640 medium (Gibco, Thermo Fisher Scientific, No. 11875093) containing 10% FBS (Gibco, Thermo Fisher Scientific, No. 16000044) and 1% penicillin–streptomycin (Gibco, Thermo Fisher Scientific, No. 15070063). Raji B cells were transduced with VSV-G pseudotyped LNC-CD169 mutant retroviral vectors, followed by 1 mg mL^−1^ G418 (Gibco, Thermo Fisher Scientific) selection, as described previously.^57^ CD169^+^ Raji B cells were cultured with addition of antibiotic G418, while CD169^−^ Raji B cells were cultured in the same medium without G418. All cells were cultured at 37 °C in a humidified atmosphere containing 5% CO_2_.

#### Characterization of GM3-CD169-mediated binding of GM3-LNPs to CD169^+^ Raji B Cells

5 × 10^5^ CD169^+^ Raji B and CD169^−^ Raji B cells were pelleted after centrifugation (270*g*, 5 min). 2 mol% of fluorescent lipid (16 : 0 Liss Rhod PE) was incorporated into all LNPs for labelling, and the absorbance of LNPs was controlled to be the same. 30 μL LNPs with and without GM3 (in 0.1 mL of 10% FBS RPMI-1640) were added to the cell pellets and the mixture was incubated at 37 °C, 5% CO_2_ for 5 min. Unbound LNPs were washed twice with PBS by centrifugation (270*g*, 5 min), and cells were subsequently fixed with 4% paraformaldehyde (PFA) (Sigma-Aldrich, St Louis, MO) for 10 min at room temperature. Following the last washing step, the fluorescence intensity of each sample was measured by flow cytometry using a FACSCalibur cytometer (BD Biosciences, San Jose, CA) and the data was analyzed through Flowing Software 2. All fluorescence intensities were background corrected (cells without treatment were used as background), and the calculated difference was used for the analysis. GeoMean intensities for specific binding (Fig. 9) were determined by subtracting non-specific binding (binding of LNPs to CD169^−^ Raji B cells) from total binding (binding of LNPs to CD169^+^ Raji B cells).

#### Statistical analysis

All data are presented as mean ± standard deviation (SD). Data presentation and sample size for statistical analysis of individual experiments are specified in figure captions. Statistical significance of data was determined using two-sample Student's *t*-test as implemented in Origin. One asterisk (*) indicates significant differences at *p* ≤ 0.05, two asterisks (**) for *p* ≤ 0.01, three asterisks (***) for *p* ≤ 0.001, and four asterisks (****) for *p* ≤ 0.0001. NS was used to demonstrate nonsignificant differences.

## Author contributions

BMR and YG conceived and designed the project. YG performed experiments, analysed the data, and generated figures. BMR and YG wrote the manuscript. All authors assisted with the interpretation of the results and contributed to the final manuscript.

## Conflicts of interest

BMR holds patents for GM3-functionalized nanoparticles.

## Supplementary Material

NR-016-D3NR06464F-s001
