# Supplementary material for: Membrane fluidity properties of lipid-coated polylactic acid nanoparticles
Source: Nanoscale. 2024 Apr 1;16(17):8533–45. doi: 10.1039/d3nr06464f (PMC11064779; doi:10.1039/d3nr06464f)
Supplement: NR-016-D3NR06464F-s001 [file NR-016-D3NR06464F-s001.pdf]

Electronic Supplementary Material (ESI) for Nanoscale.
This journal is © The Royal Society of Chemistry 2024

Supporting Information to

Membrane fluidity properties of lipid-coated polylactic acid nanoparticles

Yuanqing Gu [a] and Björn M. Reinhard*[b]

[a]Department of Chemistry and The Photonics Center, Boston University, Boston, MA 02215, USA. Email: yqgu@bu.edu

[b]Department of Chemistry and The Photonics Center, Boston University, Boston, MA 02215, USA. Email: bmr@bu.edu

Table of Contents

S1. Quantification of relative cholesterol concentration in LNP_x^{CHOL} by cholesterol assay kit 2

S2. Colocalization of LNPs and fluorescently labelled lipid membrane around the polymer core by darkfield and fluorescence imaging 3

S3. DSC thermograms of LNP_{20}^{CHOL}. 4

S4. Comparison of C-Laurdan GP(T) measurements of LNPs and PLA NPs............5

S5. C-Laurdan GP(T) measurements of $Liposome_x^{CHOL}$ and LNP_x^{CHOL} with and without background subtraction............6

S6. The representative tangent constructions for T_{on} and T_{off} determination 7

S1. Quantification of relative cholesterol concentration in LNP_x^{CHOL} by cholesterol assay kit

Cholesterol assay. Relative cholesterol loading on LNP_x^{CHOL} were quantified through Amplex™ Red Cholesterol Assay Kit (NO. A12216, ThermoFisher). A cholesterol standard curve was prepared through diluting the appropriate amount of 2 mg mL^{-1} (5.17 mM) cholesterol reference standard into 1X Reaction Buffer to produce cholesterol concentrations of 0 to 8 µg mL^{-1} (0 to ~20 µM). 1X Reaction Buffer without cholesterol was used as a negative control. The cholesterol-containing samples were diluted in 1X Reaction Buffer. A volume of 50 µL of the diluted samples, standards, and controls were pipetted into separate wells of a 96-well microplate. Then 50 µL of the Amplex® Red reagent/HRP/cholesterol oxidase/cholesterol esterase working solution was added to each microplate well containing the samples, standards, and controls. The reactions were incubated for 1 hour at 37 °C protected from light. The fluorescence was measured in a fluorescence microplate reader (SpectraMax M5) using excitation at 560 nm and emission detection at 590 nm. The background fluorescence was corrected by subtracting the values derived from the no-cholesterol control.

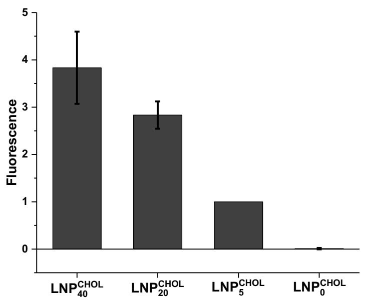

Fig. S1. Relative cholesterol concentration in LNP_x^{CHOL} quantified by cholesterol assay kit (n=3). LNP_0^{CHOL} means LNPs without cholesterol as blank controls. LNP_5^{CHOL} was used as a reference and other LNP_x^{CHOL} were normalized based on LNP_5^{CHOL} for ratiometric fluorescence in cholesterol assay. Error bars represent standard deviation.

S2. Colocalization of LNPs and fluorescently labelled lipid membrane around the polymer core by darkfield and fluorescence imaging

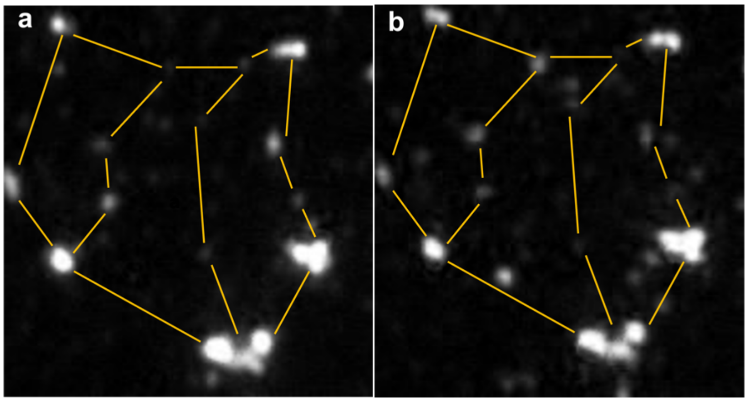

$$Colocalization\ (\%) = \frac{Number\ of\ NPs\ in\ darkfield\ image\ overlapping\ with\ fluorescence\ image}{Total\ number\ of\ NPs\ in\ darkfield\ image}$$

$$= \frac{14}{20} = 70\%$$

Fig. S2. Colocalization of LNPs and fluorescently labelled lipid membrane around the polymer core by darkfield and fluorescence imaging. One example of dark field image (a) and fluorescence image (b) of LNP_{40}^{DOPC}. 2 mol% of fluorescent lipid (16:0 Liss Rhod PE) was incorporated to lipid membrane for labelling. Yellow lines were used for colocalization between dark field and fluorescence images. Colocalization percentage was calculated by dividing the number of NPs in darkfield image overlapping with fluorescence image by the total number of NPs in darkfield image. Four image sets were used for the average colocalization percentage calculation. The colocalization percentage of the given example is 70%, while the average colocalization percentage is 73%.

S3. DSC thermograms of LNP_{20}^{CHOL}.

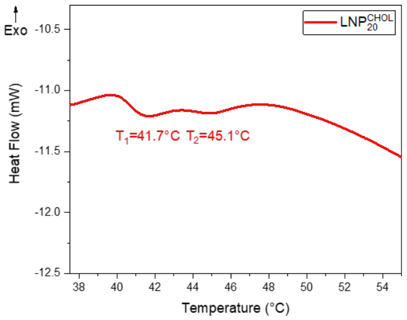

Fig. S3. DSC thermograms of LNP_{20}^{CHOL}. Two separate features, T_1 and T_2 , indicate glass and phase transition of polymer core and lipid membrane.

S4. Comparison of C-Laurdan GP(T) measurements of LNPs and PLA NPs

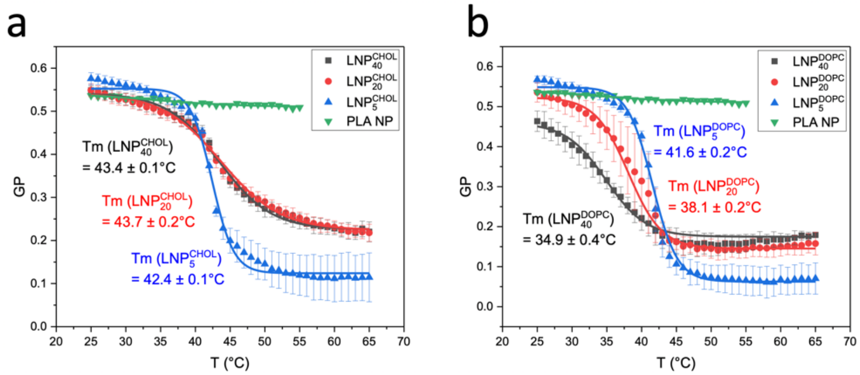

Fig. S4. Comparison of C-Laurdan GP(T) measurements of LNPs and PLA NPs. (a) GP(T) curves for LNP$_x^{CHOL}$ and PLA NP. (b) GP(T) curves for LNP$_x^{DOPC}$ and PLA NP. Error bars in (a, b) represent standard deviation.

S5. C-Laurdan GP(T) measurements of Liposome$_x^{CHOL}$ and LNP$_x^{CHOL}$ with and without background subtraction

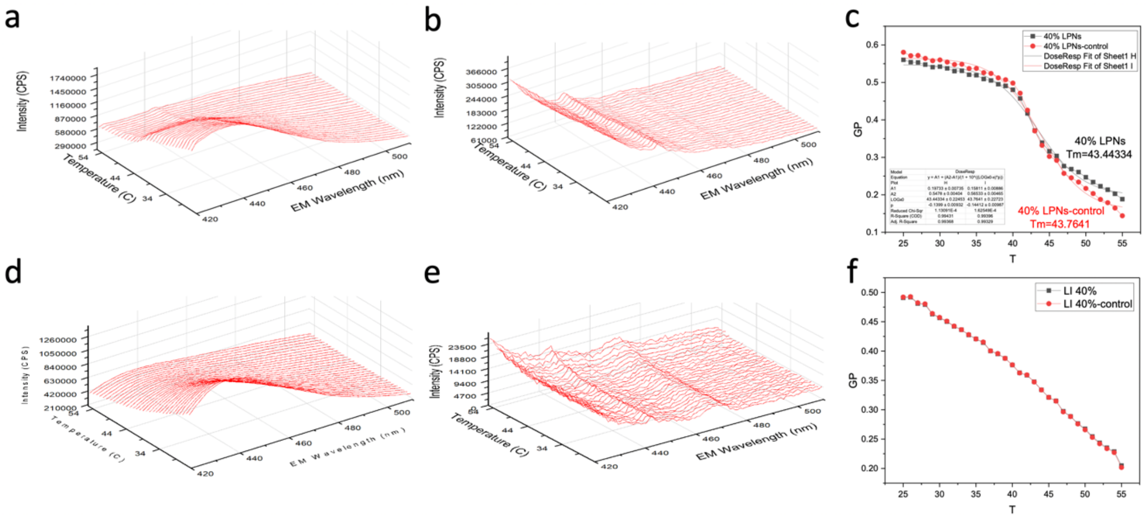

Fig. S5. C-Laurdan spectra and GP measurements of LNP$_{40}^{CHOL}$ and Liposome$_{40}^{CHOL}$ with and without C-Laurdan. 3-dimensional (3-D) spectra of LNP$_{40}^{CHOL}$ with (a) and without (b) C-Laurdan. (c) C-Laurdan GP(T) plots for LNP$_{40}^{CHOL}$. The black-square curve was obtained directly from GP values calculated from spectra (a). The red-circle curve was obtained by subtracting the GP values of spectra (b) from the GP values of spectra (a) (background correction). 3-D spectra of Liposome$_{40}^{CHOL}$ with (d) and without (e) C-Laurdan. (f) C-Laurdan GP(T) plots for Liposome$_{40}^{CHOL}$. The black-square curve was obtained directly from GP values calculated from spectra (d). The red-circle curve was obtained by subtracting the GP values of spectra (e) from the GP values of spectra (d) (background correction).

S6. The representative tangent constructions for T_{on} and T_{off} determination

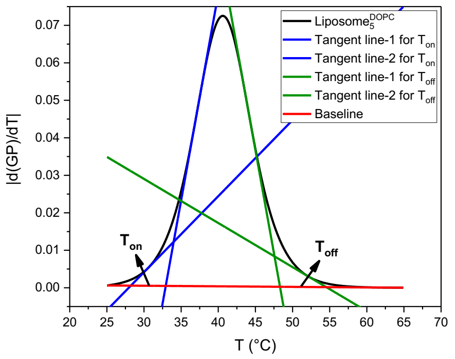

Fig. S6. The representative tangent constructions for T_{on} and T_{off} determination. For example, in the case of Liposome $_5^{DOPC}$, the two blue lines are tangent to the left side of the bell-shaped curve at the points of steepest slope and of greatest curvature. The mean of the intersection points of the two blue lines with the baseline (red line) was taken as T_{on}. Similarly, two green lines are tangent to the right side of the bell-shaped curve at the points of steepest slope and of greatest curvature. The mean of the intersection points of the two green lines with the baseline (red line) was taken as T_{off}.
